# (1*H*-1,2,3-Benzotriazol-1-yl)methyl 2,2-dimethyl­propano­ate

**DOI:** 10.1107/S1600536812010252

**Published:** 2012-03-14

**Authors:** Sen Xu, Yingzhong Shen

**Affiliations:** a Department of Applied Chemistry, School of Material Science and Engineering, Nanjing University of Aeronautics and Astronautics, Nanjing, Jiangsu Province 210016, People’s Republic of China

## Abstract

In the title compound, C_12_H_15_N_3_O_2_, the dihedral angle between the mean planes of the benzene and triazole rings is 0.331 (53) °. The side chain of the pivalate unit forms a dihedral angle of 69.04 (12)° with the benzotriazole unit. The ester group and two methyl groups of the pivalate unit are disordered with an occupancy ratio of 0.731 (3):0.269 (3). In the crystal, weak π–π stacking inter­actions are observed between inversion-related benzene rings [centroid–centroid distance = 3.9040 (1) Å].

## Related literature

For a related structure, see: Li & Chen (2011 ▶). For applications of benzotriazole derivatives, see: Wan & Lv (2010 ▶). For related coordination compounds, see: Hang & Ye (2008 ▶); Xu & Shen (2012 ▶).
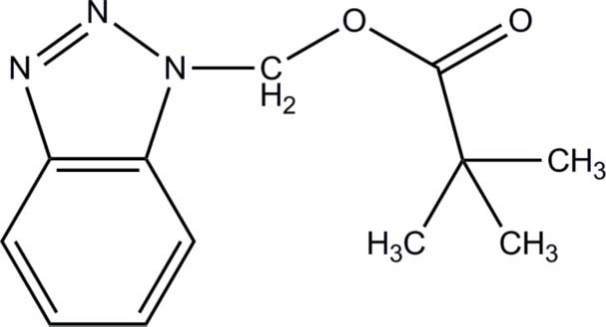


## Experimental

### 

#### Crystal data

C_12_H_15_N_3_O_2_
*M* *_r_* = 233.27
Monoclinic, 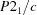

*a* = 8.1507 (3) Å
*b* = 16.7258 (8) Å
*c* = 9.2967 (4) Å
β = 98.354 (3)°
*V* = 1253.94 (9) Å^3^
*Z* = 4
Mo *K*α radiation
μ = 0.09 mm^−1^
*T* = 296 K
0.30 × 0.25 × 0.22 mm

#### Data collection

Bruker SMART CCD area-detector diffractometer
Absorption correction: multi-scan (*SADABS*; Bruker, 2001 ▶) *T*_min_ = 0.975, *T*_max_ = 0.981
9487 measured reflections
2206 independent reflections
1738 reflections with *I* > 2σ(*I*)
*R*_int_ = 0.037

#### Refinement

*R*[*F* ^2^ > 2σ(*F* ^2^)] = 0.043
*wR*(*F* ^2^) = 0.128
*S* = 1.03
2206 reflections
214 parameters
H atoms treated by a mixture of independent and constrained refinement
Δρ_max_ = 0.18 e Å^−3^
Δρ_min_ = −0.10 e Å^−3^

### 

Data collection: *SMART* (Bruker, 2007 ▶); cell refinement: *SAINT* (Bruker, 2007 ▶); data reduction: *SAINT*; program(s) used to solve structure: *SHELXS97* (Sheldrick, 2008 ▶); program(s) used to refine structure: *SHELXL97* (Sheldrick, 2008 ▶); molecular graphics: *DIAMOND* (Brandenburg, 1999 ▶); software used to prepare material for publication: *SHELXTL* (Sheldrick, 2008 ▶).

## Supplementary Material

Crystal structure: contains datablock(s) global, I. DOI: 10.1107/S1600536812010252/jj2123sup1.cif

Structure factors: contains datablock(s) I. DOI: 10.1107/S1600536812010252/jj2123Isup2.hkl

Supplementary material file. DOI: 10.1107/S1600536812010252/jj2123Isup3.cml

Additional supplementary materials:  crystallographic information; 3D view; checkCIF report

## Figures and Tables

**Fig. 1.**
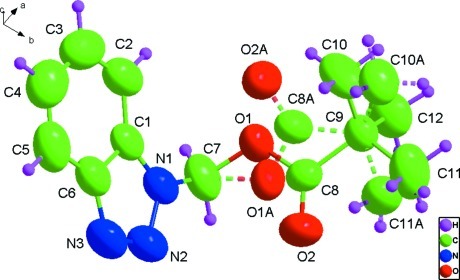
The molecular structure of the title compound with 50% probability displacement ellipsoids. Dashed lines indicate disordered ester and methyl groups.

## supplementary crystallographic information

### Comment

Nobenzotriazole derivatives have been extensively studied, not only for their potential application in antibacterial activities (Wan & Lv, 2010), but also for synthesizing benzotriazole coordination complexs (Hang & Ye, 2008). In continuing our work with new benzotriazole coordination complexs (Xu & Shen, 2012), we have synthesized a new N-donor benzotriazole derivative ligand, C_12_H_15_N_3_O_2_, Fig. 1 Bond lengths and angles are similar to those in related benzotriazol-1-yl intermediate derivatives (Li & Chen, 2011, Wan & Lv, 2010). The ester group and two methyl groups in the pivalate unit are disordered, In the crystal, weak π–π stacking interactions are abserved between the inversion related phenyl rings (centroid-centroid distances = 3.9040 (1)°).

### Experimental

To a 250 ml round flask was added (1*H*-benzo[*d*][1,2,3]triazol-1-yl)methanol(3.73 g, 0.025 mol), methylene chloride(20 mL) and triethylamine(7.0 mL) with magnetic stirring atroom tempertature for 1 h. Pivaloyl chloride(3.32 g, 0.028 mol) was then added to the solution in the ice bath. The mixture was then refluxed for 6 h at 303 K under a nitrogen atmosphere. When the reaction was completed, the solvent was evaporated *in vacuo*, and the residue was washed with distilled water and purified by recrystallization from diethyl ether (Yield: 83.2%). Colorless crystals suitable for X-ray analysis were obtained by slow evaporation from diethyl ether at room temperature.

### Refinement

The H atoms on the CH_2_ group were located by difference maps and freely refined without constraints. H atoms bonded to the remaining C atoms were included in calculated positions and treated as riding with C–H = 0.93–0.97Å and *U*_iso_(H)=1.2Ueq(aromatic C) or *U*_iso_(H) = 1.5Ueq(CH_3_). The ester(–O—CO–) and two methyl groups (C10, C11) in the pivalate unit are disordered over two side positions with site occupation factors 0.731 (3)/0.269 (3). The C—C, C—O distances and angles of the disordered groups were refined without restraints.

### Crystal data

**Table:**

C_12_H_15_N_3_O_2_	*F*(000) = 496
*M**_r_* = 233.27	*D*_x_ = 1.236 Mg m^−^^3^
Monoclinic, *P*2_1_/*c*	Mo *K*α radiation, λ = 0.71073 Å
Hall symbol: -P 2ybc	Cell parameters from 3348 reflections
*a* = 8.1507 (3) Å	θ = 2.5–26.7°
*b* = 16.7258 (8) Å	µ = 0.09 mm^−^^1^
*c* = 9.2967 (4) Å	*T* = 296 K
β = 98.354 (3)°	Block, colourless
*V* = 1253.94 (9) Å^3^	0.30 × 0.25 × 0.22 mm
*Z* = 4	

### Data collection

**Table:**

Bruker SMART CCD area-detector diffractometer	2206 independent reflections
Radiation source: fine-focus sealed tube	1738 reflections with *I* > 2σ(*I*)
Graphite monochromator	*R*_int_ = 0.037
Detector resolution: 10.0 pixels mm^-1^	θ_max_ = 25.0°, θ_min_ = 2.4°
phi and ω scans	*h* = −9→9
Absorption correction: multi-scan (*SADABS*; Bruker, 2001)	*k* = −19→19
*T*_min_ = 0.975, *T*_max_ = 0.981	*l* = −11→10
9487 measured reflections	

### Refinement

**Table:**

Refinement on *F*^2^	Secondary atom site location: difference Fourier map
Least-squares matrix: full	Hydrogen site location: inferred from neighbouring sites
*R*[*F*^2^ > 2σ(*F*^2^)] = 0.043	H atoms treated by a mixture of independent and constrained refinement
*wR*(*F*^2^) = 0.128	*w* = 1/[σ^2^(*F*_o_^2^) + (0.060*P*)^2^ + 0.1761*P*] where *P* = (*F*_o_^2^ + 2*F*_c_^2^)/3
*S* = 1.03	(Δ/σ)_max_ < 0.001
2206 reflections	Δρ_max_ = 0.18 e Å^−^^3^
214 parameters	Δρ_min_ = −0.10 e Å^−^^3^
0 restraints	Extinction correction: *SHELXL97* (Sheldrick, 2008), Fc^*^=kFc[1+0.001xFc^2^λ^3^/sin(2θ)]^-1/4^
Primary atom site location: structure-invariant direct methods	Extinction coefficient: 0.240 (12)

### Special details

**Table:**

Geometry. All e.s.d.'s (except the e.s.d. in the dihedral angle between two l.s. planes) are estimated using the full covariance matrix. The cell e.s.d.'s are taken into account individually in the estimation of e.s.d.'s in distances, angles and torsion angles; correlations between e.s.d.'s in cell parameters are only used when they are defined by crystal symmetry. An approximate (isotropic) treatment of cell e.s.d.'s is used for estimating e.s.d.'s involving l.s. planes.
Refinement. Refinement of *F*^2^ against ALL reflections. The weighted *R*-factor *wR* and goodness of fit *S* are based on *F*^2^, conventional *R*-factors *R* are based on *F*, with *F* set to zero for negative *F*^2^. The threshold expression of *F*^2^ > σ(*F*^2^) is used only for calculating *R*-factors(gt) *etc*. and is not relevant to the choice of reflections for refinement. *R*-factors based on *F*^2^ are statistically about twice as large as those based on *F*, and *R*- factors based on ALL data will be even larger.

### Fractional atomic coordinates and isotropic or equivalent isotropic displacement parameters (Å^2^)

**Table:**

	*x*	*y*	*z*	*U*_iso_*/*U*_eq_	Occ. (<1)
O1	0.2265 (3)	0.74088 (14)	0.5551 (2)	0.0766 (6)	0.731 (3)
O2	0.1680 (2)	0.82951 (11)	0.3775 (2)	0.0968 (7)	0.731 (3)
O1A	0.1824 (8)	0.7730 (4)	0.4914 (9)	0.0822 (19)	0.269 (3)
O2A	0.3972 (6)	0.6944 (3)	0.5729 (7)	0.111 (2)	0.269 (3)
N1	0.04557 (15)	0.65313 (9)	0.40894 (14)	0.0722 (4)	
N2	−0.05972 (18)	0.66728 (11)	0.28420 (19)	0.0925 (5)	
N3	−0.0594 (2)	0.60557 (12)	0.19956 (17)	0.0958 (5)	
C1	0.11500 (17)	0.57919 (10)	0.40336 (15)	0.0648 (4)	
C2	0.2279 (2)	0.53528 (13)	0.49892 (19)	0.0840 (6)	
H2	0.2739	0.5551	0.5893	0.101*	
C3	0.2672 (2)	0.46083 (15)	0.4516 (3)	0.1008 (7)	
H3	0.3428	0.4297	0.5118	0.121*	
C4	0.1986 (3)	0.43031 (14)	0.3178 (3)	0.1009 (7)	
H4	0.2283	0.3793	0.2915	0.121*	
C5	0.0898 (3)	0.47290 (13)	0.2249 (2)	0.0927 (6)	
H5	0.0446	0.4524	0.1349	0.111*	
C6	0.04751 (19)	0.54945 (11)	0.26945 (18)	0.0744 (5)	
C7	0.0640 (2)	0.71089 (15)	0.5249 (3)	0.0871 (6)	
H1M	−0.025 (3)	0.7513 (14)	0.502 (2)	0.120 (8)*	
H2M	0.055 (3)	0.6865 (14)	0.618 (3)	0.127 (8)*	
C8	0.2642 (4)	0.80275 (16)	0.4734 (3)	0.0621 (6)	0.731 (3)
C9	0.44199 (18)	0.83291 (9)	0.52164 (16)	0.0637 (4)	
C10	0.5617 (4)	0.7648 (2)	0.5330 (4)	0.1025 (11)	0.731 (3)
H10A	0.5324	0.7265	0.6017	0.154*	0.731 (3)
H10B	0.6717	0.7844	0.5650	0.154*	0.731 (3)
H10C	0.5581	0.7399	0.4396	0.154*	0.731 (3)
C11	0.4801 (6)	0.8957 (3)	0.4141 (4)	0.1158 (13)	0.731 (3)
H11A	0.4703	0.8725	0.3187	0.174*	0.731 (3)
H11B	0.5910	0.9151	0.4419	0.174*	0.731 (3)
H11C	0.4031	0.9392	0.4135	0.174*	0.731 (3)
C12	0.4501 (3)	0.87363 (13)	0.6689 (2)	0.0948 (6)	
H12A	0.3671	0.9147	0.6633	0.142*	
H12B	0.5578	0.8970	0.6956	0.142*	
H12C	0.4304	0.8349	0.7407	0.142*	
C8A	0.3453 (9)	0.7596 (5)	0.5306 (7)	0.0724 (18)	0.269 (3)
C10A	0.6271 (11)	0.8008 (6)	0.4959 (10)	0.093 (3)	0.269 (3)
H10D	0.6735	0.7684	0.5770	0.139*	0.269 (3)
H10E	0.6983	0.8458	0.4873	0.139*	0.269 (3)
H10F	0.6172	0.7696	0.4085	0.139*	0.269 (3)
C11A	0.3845 (15)	0.8843 (7)	0.3900 (12)	0.111 (3)	0.269 (3)
H11D	0.3597	0.8509	0.3057	0.166*	0.269 (3)
H11E	0.4705	0.9214	0.3756	0.166*	0.269 (3)
H11F	0.2868	0.9132	0.4052	0.166*	0.269 (3)

### Atomic displacement parameters (Å^2^)

**Table:**

	*U*^11^	*U*^22^	*U*^33^	*U*^12^	*U*^13^	*U*^23^
O1	0.0696 (14)	0.0778 (14)	0.0794 (12)	−0.0172 (11)	0.0009 (9)	0.0118 (10)
O2	0.0858 (12)	0.0929 (13)	0.1025 (14)	0.0056 (10)	−0.0178 (10)	0.0215 (11)
O1A	0.062 (4)	0.068 (4)	0.118 (5)	−0.009 (3)	0.017 (3)	0.009 (3)
O2A	0.076 (3)	0.093 (4)	0.156 (5)	−0.011 (3)	−0.008 (3)	0.044 (4)
N1	0.0554 (7)	0.0852 (10)	0.0737 (8)	−0.0143 (7)	0.0016 (6)	0.0047 (7)
N2	0.0710 (9)	0.1006 (12)	0.0981 (12)	−0.0067 (8)	−0.0137 (8)	0.0126 (10)
N3	0.0864 (10)	0.1078 (13)	0.0844 (10)	−0.0175 (9)	−0.0168 (8)	0.0037 (10)
C1	0.0488 (7)	0.0823 (11)	0.0636 (9)	−0.0170 (7)	0.0091 (6)	0.0083 (8)
C2	0.0680 (10)	0.1065 (15)	0.0754 (10)	−0.0131 (10)	0.0034 (8)	0.0167 (10)
C3	0.0809 (12)	0.1038 (16)	0.1190 (17)	0.0079 (11)	0.0189 (12)	0.0314 (14)
C4	0.0994 (15)	0.0939 (15)	0.1166 (17)	−0.0059 (12)	0.0396 (13)	0.0078 (14)
C5	0.0960 (13)	0.1002 (15)	0.0861 (12)	−0.0315 (12)	0.0270 (11)	−0.0105 (12)
C6	0.0626 (9)	0.0894 (12)	0.0709 (10)	−0.0198 (8)	0.0083 (7)	0.0050 (9)
C7	0.0694 (11)	0.0980 (14)	0.0957 (14)	−0.0240 (11)	0.0185 (9)	−0.0136 (12)
C8	0.0670 (15)	0.0573 (14)	0.0606 (13)	0.0068 (15)	0.0046 (13)	0.0034 (12)
C9	0.0650 (9)	0.0635 (9)	0.0618 (9)	−0.0068 (7)	0.0064 (6)	−0.0023 (7)
C10	0.0652 (17)	0.106 (3)	0.134 (3)	0.0108 (16)	0.0070 (16)	−0.040 (2)
C11	0.133 (3)	0.126 (3)	0.091 (2)	−0.052 (3)	0.025 (2)	0.0067 (19)
C12	0.1017 (14)	0.1004 (14)	0.0820 (12)	−0.0055 (11)	0.0122 (10)	−0.0232 (11)
C8A	0.060 (4)	0.082 (5)	0.074 (4)	−0.001 (4)	0.003 (3)	0.017 (4)
C10A	0.079 (5)	0.093 (6)	0.109 (6)	−0.010 (4)	0.024 (4)	−0.003 (5)
C11A	0.107 (7)	0.100 (7)	0.114 (7)	−0.021 (6)	−0.022 (6)	0.040 (5)

### Geometric parameters (Å, º)

**Table:**

O1—C8	1.345 (4)	C8—C9	1.539 (4)
O1—C7	1.406 (3)	C9—C8A	1.466 (8)
O2—C8	1.186 (3)	C9—C10	1.493 (3)
O1A—C8A	1.344 (10)	C9—C11	1.512 (4)
O1A—C7	1.481 (8)	C9—C11A	1.513 (9)
O2A—C8A	1.215 (10)	C9—C12	1.522 (2)
N1—N2	1.3594 (19)	C9—C10A	1.651 (9)
N1—C1	1.364 (2)	C10—H10A	0.9600
N1—C7	1.439 (2)	C10—H10B	0.9600
N2—N3	1.298 (2)	C10—H10C	0.9600
N3—C6	1.378 (2)	C11—H11A	0.9600
C1—C6	1.379 (2)	C11—H11B	0.9600
C1—C2	1.392 (2)	C11—H11C	0.9600
C2—C3	1.374 (3)	C12—H12A	0.9600
C2—H2	0.9300	C12—H12B	0.9600
C3—C4	1.385 (3)	C12—H12C	0.9600
C3—H3	0.9300	C10A—H10D	0.9600
C4—C5	1.348 (3)	C10A—H10E	0.9600
C4—H4	0.9300	C10A—H10F	0.9600
C5—C6	1.404 (3)	C11A—H11D	0.9600
C5—H5	0.9300	C11A—H11E	0.9600
C7—H1M	0.99 (2)	C11A—H11F	0.9600
C7—H2M	0.97 (2)		
			
C8—O1—C7	116.6 (3)	C11—C9—C11A	30.7 (3)
C8A—O1A—C7	118.3 (8)	C8A—C9—C12	106.0 (3)
N2—N1—C1	109.84 (14)	C10—C9—C12	109.57 (19)
N2—N1—C7	120.41 (17)	C11—C9—C12	107.3 (2)
C1—N1—C7	129.67 (16)	C11A—C9—C12	116.2 (5)
N3—N2—N1	108.78 (15)	C8A—C9—C8	41.5 (3)
N2—N3—C6	108.23 (14)	C10—C9—C8	110.42 (19)
N1—C1—C6	104.36 (14)	C11—C9—C8	108.1 (2)
N1—C1—C2	133.91 (16)	C11A—C9—C8	77.4 (4)
C6—C1—C2	121.73 (18)	C12—C9—C8	108.94 (14)
C3—C2—C1	115.92 (18)	C8A—C9—C10A	104.2 (4)
C3—C2—H2	122.0	C10—C9—C10A	33.0 (3)
C1—C2—H2	122.0	C11—C9—C10A	81.4 (4)
C2—C3—C4	122.6 (2)	C11A—C9—C10A	104.5 (6)
C2—C3—H3	118.7	C12—C9—C10A	110.8 (4)
C4—C3—H3	118.7	C8—C9—C10A	133.9 (4)
C5—C4—C3	121.5 (2)	C9—C10—H10A	109.5
C5—C4—H4	119.2	C9—C10—H10B	109.5
C3—C4—H4	119.2	C9—C10—H10C	109.5
C4—C5—C6	117.38 (19)	C9—C11—H11A	109.5
C4—C5—H5	121.3	C9—C11—H11B	109.5
C6—C5—H5	121.3	C9—C11—H11C	109.5
N3—C6—C1	108.78 (17)	C9—C12—H12A	109.5
N3—C6—C5	130.39 (18)	C9—C12—H12B	109.5
C1—C6—C5	120.82 (18)	H12A—C12—H12B	109.5
O1—C7—N1	112.46 (17)	C9—C12—H12C	109.5
O1—C7—O1A	33.7 (2)	H12A—C12—H12C	109.5
N1—C7—O1A	108.3 (3)	H12B—C12—H12C	109.5
O1—C7—H1M	115.9 (14)	O2A—C8A—O1A	121.4 (8)
N1—C7—H1M	107.8 (13)	O2A—C8A—C9	127.2 (6)
O1A—C7—H1M	87.4 (14)	O1A—C8A—C9	111.3 (7)
O1—C7—H2M	99.3 (14)	C9—C10A—H10D	109.5
N1—C7—H2M	111.8 (14)	C9—C10A—H10E	109.5
O1A—C7—H2M	128.2 (14)	H10D—C10A—H10E	109.5
H1M—C7—H2M	109.4 (18)	C9—C10A—H10F	109.5
O2—C8—O1	122.3 (4)	H10D—C10A—H10F	109.5
O2—C8—C9	125.9 (3)	H10E—C10A—H10F	109.5
O1—C8—C9	111.7 (2)	C9—C11A—H11D	109.5
C8A—C9—C10	73.1 (3)	C9—C11A—H11E	109.5
C8A—C9—C11	141.4 (3)	H11D—C11A—H11E	109.5
C10—C9—C11	112.4 (3)	C9—C11A—H11F	109.5
C8A—C9—C11A	114.5 (5)	H11D—C11A—H11F	109.5
C10—C9—C11A	127.8 (6)	H11E—C11A—H11F	109.5
			
C1—N1—N2—N3	−0.27 (18)	C7—O1—C8—O2	−2.4 (4)
C7—N1—N2—N3	−177.51 (15)	C7—O1—C8—C9	177.12 (17)
N1—N2—N3—C6	0.17 (19)	O2—C8—C9—C8A	−157.5 (5)
N2—N1—C1—C6	0.25 (16)	O1—C8—C9—C8A	23.0 (4)
C7—N1—C1—C6	177.16 (14)	O2—C8—C9—C10	−130.2 (3)
N2—N1—C1—C2	−179.74 (16)	O1—C8—C9—C10	50.3 (3)
C7—N1—C1—C2	−2.8 (3)	O2—C8—C9—C11	−6.9 (3)
N1—C1—C2—C3	179.88 (16)	O1—C8—C9—C11	173.7 (3)
C6—C1—C2—C3	−0.1 (2)	O2—C8—C9—C11A	−4.3 (6)
C1—C2—C3—C4	−0.5 (3)	O1—C8—C9—C11A	176.3 (6)
C2—C3—C4—C5	0.8 (3)	O2—C8—C9—C12	109.4 (2)
C3—C4—C5—C6	−0.5 (3)	O1—C8—C9—C12	−70.0 (2)
N2—N3—C6—C1	−0.02 (19)	O2—C8—C9—C10A	−102.3 (5)
N2—N3—C6—C5	179.30 (17)	O1—C8—C9—C10A	78.2 (5)
N1—C1—C6—N3	−0.14 (16)	C7—O1A—C8A—O2A	10.7 (10)
C2—C1—C6—N3	179.85 (14)	C7—O1A—C8A—C9	−166.6 (4)
N1—C1—C6—C5	−179.54 (13)	C10—C9—C8A—O2A	17.8 (7)
C2—C1—C6—C5	0.5 (2)	C11—C9—C8A—O2A	122.8 (7)
C4—C5—C6—N3	−179.41 (17)	C11A—C9—C8A—O2A	142.3 (9)
C4—C5—C6—C1	−0.2 (2)	C12—C9—C8A—O2A	−88.2 (7)
C8—O1—C7—N1	85.2 (3)	C8—C9—C8A—O2A	171.1 (10)
C8—O1—C7—O1A	−4.4 (4)	C10A—C9—C8A—O2A	28.7 (9)
N2—N1—C7—O1	−118.0 (2)	C10—C9—C8A—O1A	−165.1 (6)
C1—N1—C7—O1	65.3 (3)	C11—C9—C8A—O1A	−60.2 (8)
N2—N1—C7—O1A	−82.3 (3)	C11A—C9—C8A—O1A	−40.7 (9)
C1—N1—C7—O1A	101.1 (4)	C12—C9—C8A—O1A	88.8 (6)
C8A—O1A—C7—O1	17.8 (4)	C8—C9—C8A—O1A	−11.8 (4)
C8A—O1A—C7—N1	−85.5 (6)	C10A—C9—C8A—O1A	−154.2 (6)
